# The validity and reliability of the English version of the diabetes distress scale for type 2 diabetes patients in Malaysia

**DOI:** 10.1186/s12875-017-0601-9

**Published:** 2017-02-20

**Authors:** Ying Woei Chin, Pauline Siew Mei Lai, Yook Chin Chia

**Affiliations:** 1 Department of Primary Care Medicine, University of Malaya Primary Care Research Group, Faculty of Medicine, University of Malaya, 50603 Kuala Lumpur, Malaysia; 2 Sunway lnstitute for Healthcare Development, Sunway University, Bandar Sunway, Selangor Malaysia

**Keywords:** Diabetes Distress Scale, DDS-17, Validity, Reliability, Malaysia, Type 2 diabetes, English

## Abstract

**Background:**

Several disease specific instruments have been developed to identify and assess diabetes distress. In Malaysia, the Problem Areas in Diabetes Scale has been validated in Malay, but it does not have specific domains to assess the different areas of diabetes-related distress. Hence, we decided to use the Diabetes Distress Scale instead. To date, only the Malay version of the Diabetes Distress Scale has been validated in Malaysia. However, English is widely spoken by Malaysians, and is an important second language in Malaysia. Therefore, our aim was to determine the validity and reliability of the English version of the Diabetes Distress Scale among patients with type 2 diabetes in Malaysia.

**Methods:**

The Diabetes Distress Scale was administered to 114 patients with type 2 diabetes, who could understand English, at baseline and 4 weeks later, at a primary care clinic in Malaysia. To assess for convergent validity, the Depression Anxiety Stress Scale was administered at baseline. Discriminative validity was assessed by analysing the total diabetes distress scores of participants with poor (Hb_A1c_ > 7.0%) and good glycaemic control (Hb_A1c_ ≤ 7.0%).

**Results:**

The majority of our participants were male 65(57.0%), with a median duration of diabetes of 9.5 years. Exploratory factor analysis showed that the Diabetes Distress Scale had 4 subscales, as per the original Diabetes Distress Scale. The overall Cronbach’s α was 0.920 (range = 0.784–0.859 for each subscale). The intraclass correlation ranged from 0.436 to 0.643 for test-retest. The Diabetes Distress Scale subscales were significantly correlated with the different subscales of the Depression Anxiety Stress Scale (spearman’s rho range = 0.427–0.509, *p* < 0.001). Patients with poor glycaemic control had significantly higher diabetes distress score (1.88) compared to those with good glycaemic control (1.50) (*p* < 0.001).

**Conclusions:**

The English version of the Diabetes Distress Scale was found to be a valid and reliable instrument to evaluate diabetes distress among patients with type 2 diabetes in Malaysia. It can be used in clinical practice to offer a useful indicator of the effect of diabetes-induced distress during clinic visits, especially for patients with poor glycemic control. This would ensure that these patients are provided the care that is required.

**Electronic supplementary material:**

The online version of this article (doi:10.1186/s12875-017-0601-9) contains supplementary material, which is available to authorized users.

## Background

Living with diabetes can be difficult, as it is can affect the patient physically as well as psychologically [1, 2]. In addition to having to take medications routinely, and having to see their doctor regularly, patients with diabetes often need to make several major lifestyle changes to achieve good glycemic control to avoid long-term complications [3]. This requires mental preparedness of change, self-care and discipline from the patient; as well as support from family, friends and health care personnel [4]. As a consequence, patients may sometimes feel frustrated, overwhelmed or discouraged. These emotional burdens and worries about diabetes, and its management, threats of complications, and unmet needs of moral support from family, friends and health care providers have been recognized as diabetes distress [1]. Diabetes distress is on the rise as a result of the higher global burden of diabetes. In the United States, the prevalence of diabetes distress ranged from 21.0 to 65.5% [5–7], whilst in the Netherlands it was 8.8% [8]. In Asia, the prevalence of diabetes distress was 64% in China [9], 35% in Iran [10], 48.5% in Bangladesh [11], and 11–12.5% in Malaysia [12, 13]. Significant factors related to lower diabetes distress were older age, lower body mass index, higher self-efficacy, higher levels of health care provider support, and a healthy diet [14].

Although studies have shown that emotional problems are common among patients with diabetes, diabetes distress remains largely undetected [15]. Most patients with diabetes do not voice their emotional problems, or seek help for the distress they experience [15]. If diabetes distress is left untreated, it may affect self-management, and lead to poor health outcomes [16, 17]. It is therefore important to identify patients at a primary care level with diabetes distress, in view of its high correlation with depression [9, 10, 18].

Several disease specific instruments have been developed to identify and assess diabetes distress: the Diabetes Distress Scale (DDS-17) [19], the Diabetes Health Profile (DHP-18) [20], the Diabetes-specific Quality-of-Life Scale (DSQOLS) [21], the Questionnaire on Stress in patients with Diabetes (QSD) [22] and the Problem Areas in Diabetes Scale (PAID) [23]. In Malaysia, the PAID has been validated in Malay to assess diabetic-related emotional distress [24]. However, the PAID does not have specific domains to assess the different areas of diabetes-related distress, as there is only one item that explored the patient’s view on their health care providers [19]. In addition, some items (e.g., “not having clear and concrete goals for your diabetes care”) were difficult to understand [19]. Hence, we decided to use the DDS-17 instead. To date, only the Malay version of the DDS-17 has been validated in Malaysia [25]. We decided to validate the DDS-17 in English, as English is an important second language in Malaysia, and is widely spoken in countries which were ex-colonies of the United Kingdom [26]. Therefore, the aim of our study was to determine the validity and reliability of the English version of the DDS-17, so that diabetes distress can be assessed among patients who can only understand English in Malaysia.

## Methods

This validation study was conducted in a government primary care clinic, located within a tertiary hospital in Kuala Lumpur, Malaysia, from October to November 2014.

### Participants

Included were patients who had been diagnosed with type 2 diabetes for at least a year, who were on regular follow-up, aged 18 years and above, and who were able to understand English. We left it to the participants themselves to decide whether they were comfortable in answering the instrument in English. If they experienced any difficulty in answering the instrument in English, then they were excluded from the study. In addition, we also excluded patients who were pregnant or breastfeeding an infant, or those with severe health or psychiatric/psychological problems that could cause cognitive impairments.

### Sample size

Sample size was calculated based on the number of items in the DDS-17 to participant ratio of 1:5.[27, 28] The DDS-17 has 17 items. Hence, the minimum number of participants required was 17*5 = 85.

### Instruments used

#### Baseline demographic questionnaire

A baseline demographic questionnaire was used to collect the demographic data of participants [such as age, gender, occupation, education level, duration of diabetes, presence of comorbidity, family history and diabetes medication(s)].

### The Diabetes Distress Scale (DDS-17)

The DDS-17 consists of 17 items with four subscales: emotional burden (5 items), physician distress (4 items), regimen distress (5 items) and interpersonal distress (3 items). Response to each item was based on a 6-point Likert scale, rated from 1 (not a problem) to 6 (a very serious problem) concerning diabetes for the past 1 month (Additional file 1). The total mean item score was calculated by summing up the responses to all items and dividing by 17. The mean score of each subscale was calculated by summing up the responses to all the items in that subscale, and dividing by the number of items. A higher score indicates higher distress level. A score of <2.0 was considered as “little or no distress”, 2.0–2.9 was considered as “moderate distress”, and ≥3.0 was considered “high distress” (i.e., a level of distress worthy of clinical attention) [5]. Permission to use the DDS-17 was obtained via personal communication (dated 24 May 2014).

### Face and content validity

The content validity of the DDS-17 was assessed by an expert panel (consisting of a family physician, a researcher who was familiar with the validation of instruments, and a family medicine trainee). The expert panel deemed that the DDS-17 was suitable to be used in its original form. Hence, no changes were made to the instrument.

The DDS-17 was then piloted in five adults with Type 2 Diabetes, from a primary care clinic, to assess for face validity. Participants were invited to read the questions, and to evaluate verbally if the items were difficult for them to comprehend. No further changes were made since no problems were reported. These participants were not included in the validation study.

### The Depression Anxiety Stress Scale (DASS-21)

The Depression Anxiety Stress Scale (DASS-21) is a generic instrument that has been used to assess the level of depression, anxiety and stress. It contains 21 items, and has three subscales: depression (7 items), anxiety (7 items) and stress (7 items), and has been validated in English in Malaysia [29–31]. Response to each item is based on a 4-point Likert scale: 0 means “did not apply to me”, 1 means “applied to me to some degree, or some of the time”, 2 means “applied to me to a considerable degree, or a good part of time” and 3 means “applied to me very much, or most of the time”. For each subscale, the score of each item was multiplied by two and summed [32]. The maximum score of the DASS-21 is 42 [33].

### Data collection

Convenience sampling was used to recruit participants, as participants had to be screened as to whether they could answer the questionnaire in English. Once these participants were identified, the purpose of the study was explained to them. For those who agreed to participate, written informed consent was obtained. Participants were then asked to fill the baseline demographic questionnaire, the DDS-17 and the DASS-21. This took approximately 15–20 min. The researcher then checked the questionnaire to ensure that all questions were answered. Four weeks later, the DDS-17 was mailed to each participant, with a postage-paid return envelope. If a reply was not obtained within a week, participants were contacted via the phone, and reminded to send in their completed DDS-17 form as soon as possible. Participants were also questioned if any significant changes or events had occurred within the past month, and all changes were documented. Medical records were reviewed to obtain the latest haemoglobinA1c (Hb_A1c_) results. Ethics approval was obtained from the University Malaya Medical Centre Medical Ethics Committee (approval no: 20147–394) prior to the commencement of the study.

### Data analysis

Data were analyzed using the Statistical Package for Social Sciences 22.0 software (Chicago, Illinois, USA). Descriptive statistics were used to describe demographic and disease characteristics of the patients and their diabetes distress scores. Percentages and frequencies were used for categorical variables, while median and interquartile ranges were calculated for continuous variables. Since data was not normally distributed, non-parametric tests were used.

### Validity

#### Factor analysis

The dimensionality of the DDS-17 was analyzed using exploratory factor analysis, using principal axis factoring and promax oblique rotation, as the factors were correlated [34]. An eigenvalue >1 on scree plots, indicates that there are more than one component in the instrument [34]. Items were then screened to identify those with factor loading >0.4 [35].

### Convergent validity

The DASS-21 measures self-rated symptoms of depression and anxiety during the past week, and has been used among people with diabetes [29]. Previous studies have shown that depressive symptoms experienced by patients with diabetes were significantly related to their anxiety and stress [9]. Hence, the total score of the DDS-17 was compared to the score of each subscale in the DASS-21. Correlations were calculated using Spearman’s rho correlation coefficient: values <0.30 was negligible, 0.30–0.49 was low, 0.50–0.69 was moderate, 0.70–0.89 was high, and 0.90–1.00 was very high [36].

### Discriminative validity

Previous literature showed that patients who had good glycaemic control have a lower diabetes distress score compared to those who had poor glycaemic control [25]. Hence, the Mann-Whitney *U*-test was used to determine whether the DDS-17 was able to discriminate between patients with good (Hb_A1c_ ≤ 7.0%) and poor glycaemic control (Hb_A1c_ > 7.0%). A *p*-value < 0.05 was considered as statistically significant.

### Reliability

#### Internal consistency

Internal consistency was assessed using Cronbach’s α coefficient to determine whether all items in a multi-item scale measured the same concept. This was calculated for the entire instrument, and for each subscale. Cronbach alpha value of <0.70 cannot be said to have adequate internal consistency, >0.70 has adequate internal consistency [37].

The corrected item-total correlation was also performed. Corrected item-total correlations were first used to identify items which did not agree well with other items in the questionnaire. Item-total correlations should be >0.4 to be considered acceptable [38]. The effect of removing a single item on the Cronbach’s α was also determined.

### Stability

The intra-class correlation coefficient (ICC) was used to analyse responses obtained at test and retest. ICC values ≥0.75 indicates excellent agreement, 0.60–0.74 shows good agreement; 0.40–0.59 indicates fair to moderate agreement, and <0.40 indicates poor agreement [39].

## Results

**Fig. 1:**
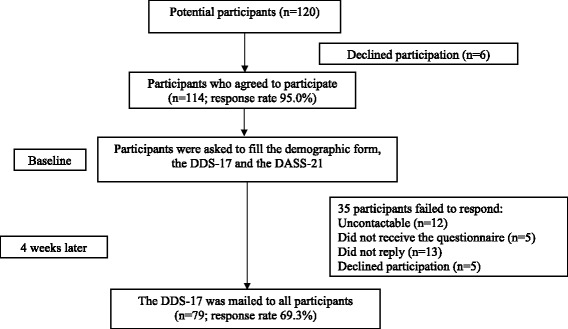
Flow of participants. DDS-17 = Diabetes Distress Scale. DASS-21 = Depression Anxiety Stress Scale

A total of 120 diabetic patients were approached, of which 114 agreed to participate (response rate = 95.0%) [Fig. 1]. The demographic characteristics of participants are shown in Table 1. There were only three participants that were exclusively on insulin treatment.

**Table 1:** Demographic characteristics of participants

Variables	Participants (*n* = 114)n(%)
Median age (years) [interquartile range^a^]	62.0 [53.0–67.0]
Gender	
Male	65 (57.0)
Female	49 (43.0)
Ethnicity	
Malay	32 (28.1)
Chinese	36 (31.5)
Indian	46 (40.4)
Presence of diabetes-related co-morbidities^b^	19 (16.7)
Positive family history of diabetes	88 (77.2)
Level of education	
Primary (6 years of education)	2 (1.8)
Secondary (12 years of education)	62 (54.4)
Tertiary (at least 16 years of education)	50 (43.8)
Median duration of diabetes (years) [IQR]	9.5 [4.0–15.0]
Median Hb_A1c_^c^ [IQR]	7.1 [6.4–8.5]
≤ 7.0	50 (47.2)
> 7.0	56 (52.8)
Management of diabetes	
Diet control	6 (5.3)
Oral hypoglycemic agent	82 (71.9)
Insulin	3 (2.6)
Combination of oral hypoglycemic agent and insulin	23 (20.2)

^a^IQR = interquartile range from the first quartile to the third quartile

^b^hypertension, dyslipidaemia, cardiovascular disease, stroke, diabetes-related eye disease and diabetes-related kidney disease

^c^glycosylated hemoglobin, only 106 patients had documented Hb_A1c_ level

### Construct validity

#### Factor analysis

Exploratory factor analysis extracted 4 factors from the DDS-17, which explained 60% of the variation. However, three items (items no. 7, 10 and 15) did not fit into the 4-factor model. Item 7 was allocated to the regime distress subscale instead of the emotional burden subscale; item 10 was cross-loaded into two subscales: the emotional burden subscale as well as the interpersonal distress subscale; whilst item 15 had a low factor loading of 0.375 (Table 2).

**Table 2:** Exploratory factor analysis of the English Diabetes Distress Scale-17 in Malaysia

Item no.	Items	Subscale	Factor loadings			
			1	2	3	4
8	Feeling that I am often failing with my diabetes routine	Regime distress	0.85			
6	Feeling that I am not testing my blood sugars frequently enough		0.76			
16	Not feeling motivated to keep up my diabetes self-management		0.69			
12	Feeling that I am not sticking closely enough to a good meal plan		0.45			
3	Not feeling confident in my day-to-day ability to manage diabetes		0.40			
7^a^	Feeling that I will end up with serious long-term complications, no matter what I do	Emotional burden	0.69			
4	Feeling angry, scared and/or depressed when I think about living with diabetes			0.80		
2	Feeling that diabetes is taking up too much of my mental and physical energy every day			0.70		
14	Feeling overwhelmed by the demands of living with diabetes			0.59		
10^a^	Feeling that diabetes controls my life			0.43		0.43
1	Feeling that my doctor doesn’t know enough about diabetes and diabetes care	Physician distress			0.82	
5	Feeling that my doctor doesn’t give me clear enough directions on how to manage my diabetes				0.80	
11	Feeling that my doctor doesn’t take my concerns seriously enough				0.71	
15^a^	Feeling that I don’t have a doctor who I can see regularly enough about my diabetes				0.38	
9	Feeling that friends or family are not supportive enough of self-care efforts (e.g., planning activities that conflict with my schedule, encouraging me to eat the “wrong” foods)	Interpersonal distress				0.95
17	Feeling that friends or family don’t give me the emotional support that I would like					0.70
13	Feeling that friends or family don’t appreciate how difficult living with diabetes can be					0.55

^a^Items that did not fit into the original 4-factor model

### Convergent validity

The total score of the DDS-17 was found to be significantly correlated with the different subscales in the DASS-21 (spearman’s rho range: 0.427–0.509, *p* < 0.001).

### Discriminative validity

Patients with poor glycaemic control had significantly higher diabetic distress than patients with good glycaemic control in all subscales and the total score (Table 3). The only exception was the interpersonal distress subscale.

**Table 3:** Diabetes distress score among patients with good and poor diabetic control

		Good control [Hb_A1c_^1^ ≤ 7.0%] (*n* = 50)		Poor control [Hb_A1c_^1^ > 7.0%] (*n* = 56)		Mann-Whitney *U* test
		Median	IQR	Median	IQR	*p*-value
Total score of the DDS-17		1.50	0.76	1.88	0.87	<0.001*
DDS-17 subscales	Emotional Burden	1.60	1.20	2.00	1.15	0.003*
	Regime Distress	1.60	1.05	2.20	1.20	0.001*
	Physician Distress	1.00	0.75	1.75	1.25	0.004*
	Interpersonal Distress	1.33	1.00	1.50	1.25	0.069

Hb_A1c_ = glycosylated hemoglobin; IQR = interquartile range; *statistically significant at *p* < 0.05; A higher score indicates higher distress level

### Reliability

The overall Cronbach’s α for the DDS-17 was 0.920, whilst the Cronbach’s α values for the subscales ranged from 0.784 to 0.859. All 17 items had a corrected item-total correlation values of >0.4. All items had fair to good ICC values (0.436–0.643, *p* < 0.001), except for item 9 (0.382) [Table 4].

**Table 4:** The psychometric properties of the Diabetes Distress Scale

		Test (*n* = 114)					Retest (*n* = 79)		
Subscales	Item no.	Cronbach’s α (*n* = 114)	Corrected item-total correlation	Cronbach’s α if item deleted	Median	IQR	Median	IQR	Intraclass correlation coefficient*
Emotional Burden	2	0.86	0.68	0.83	2.00	2.00	2.00	2.00	0.52
	4		0.76	0.81	2.00	1.00	1.00	2.00	0.61
	7		0.60	0.86	2.00	1.00	2.00	2.00	0.59
	10		0.62	0.84	2.00	2.00	2.00	2.00	0.62
	14		0.74	0.82	2.00	1.00	2.00	2.00	0.56
Physician Distress	1	0.78	0.62	0.71	1.00	1.00	1.00	1.00	0.44
	5		0.70	0.67	1.00	1.00	1.00	1.00	0.64
	11		0.59	0.73	1.00	1.00	1.00	1.00	0.52
	15		0.46	0.80	1.00	1.00	2.00	2.00	0.64
Regime Distress	3	0.84	0.54	0.83	2.00	1.00	2.00	2.00	0.49
	6		0.61	0.81	2.00	2.00	2.00	2.00	0.55
	8		0.75	0.77	2.00	2.00	2.00	2.00	0.57
	12		0.57	0.82	2.00	1.00	2.00	1.00	0.54
	16		0.73	0.78	2.00	2.00	2.00	2.00	0.48
Interpersonal Distress	9	0.81	0.67	0.72	1.00	1.00	1.00	1.00	0.38
	13		0.57	0.83	1.00	1.00	1.00	2.00	0.55
	17		0.73	0.64	1.00	1.00	1.00	1.00	0.44

IQR = interquartile range; *p < 0.001

A total of 35 (30.7%) participants experienced moderate to high diabetic distress. These participants were referred to a dietitian and a counsellor.

### Comparison of the diabetes distress scale in Malaysia with other validation studies

The psychometric properties of the DDS-17 validated in Malaysia were comparable to previous DDS-17 validation studies (Table 5).

**Table 5:** The Cronbach alpha values in previous DDS-17 validation studies

Subscales	Country the DDS-17 was validated in (language) / year							
	Malaysia (English) / 2016	Malaysia (Malay) / 2015 (25)	Thailand (Thai) / 2014 (43)	Denmark (Danish) / 2013 (42)	Norway (Norwegian) / 2012 (38)	Iran (Persian) / 2012 (41)	Hong Kong (Chinese) / 2011 (39)	United States (English) / 2005 (19)
Physician distress	0.78	0.82	0.85–0.96^3^	0.83–0.89^3^	0.81–0.87^3^	0.71	0.85	0.88
Emotional burden	0.86	0.86				0.81	0.87	0.88
Regime distress	0.84	0.93^1b^				0.78	0.82^1a^	0.90
Interpersonal distress	0.81					0.77		0.88
Total	0.92	0.94	0.95	0.92	0.92	−^2^	0.90	0.93

^1^The regime distress and the interpersonal distress subscale were combined as the regime-and-social support-related distress subscale^a^ / therapeutic support distress^b^

^2^Did not report the total DDS-17 Cronbach’s α value

^3^Did not report Cronbach’s α values of the individual subscales

## Discussion

Our study showed that the English version of the DDS-17 was a reliable and valid tool for assessing diabetic distress in patients with type 2 diabetes in Malaysia.

Exploratory factor analysis confirmed that the English version of the DDS-17 in Malaysia was a 4-factor model as per the original instrument [19]. This was in contrast to the Malay DDS-17 validation study in Malaysia, where the authors found that their instrument was a 3-factor model: where interpersonal distress and regime distress were merged as one factor [25]. Item 7: “Feeling that I will end up with serious long-term complications, no matter what I do”; was allocated to the subscale of regime distress instead of emotional burden, which was similar to two other studies [40, 41]. A possible explanation could be participants think that having long-term diabetes complication is a consequence of regime failure. Item 10: “Feeling that diabetes controls my life” was loaded into two subscales (emotional burden and interpersonal distress). This may be because participants perceived that diabetes affected their personal lives as well as their relationships with others [42]. As a result, a patient with diabetes may personally feel stressed (emotional burden), and their relationship with others may also suffer (interpersonal distress). Item 15: “Feeling that I don’t have a doctor who I can see regularly enough about my diabetes”; had a low factor loading. This may be due to the fact that our study was conducted in a government primary clinic, where patients were not at a liberty to select their doctor [41].

The total score of the DDS-17 had significantly moderate correlation with the subscales of the DASS-21, thus confirming the convergent validity of the DDS-17. The diabetes distress score was significantly higher in patients with poor compared to those with good Hb_A1c_ values [16, 40, 43, 44], thus confirming the discriminative validity of the DDS-17.

The DDS-17 had good internal consistency which was comparable to previous literature [19, 25, 40, 41, 43–45]. Our instrument also achieved stable reliability, and performed better than the Malay DDS-17 [25]. The only exception was item 9: “Feeling that friends or family are not supportive enough of self-care efforts” (e.g., planning activities that conflict with my schedule, encouraging me to eat the “wrong” foods), which had an ICC value of 0.382. This may be because this statement may be applicable to the participant at test, but may not be applicable at retest, as its occurrence may be intermittent.

Findings from our study suggest that the English DDS-17 is suitable for use in daily clinical practice (in Malaysia) to identify and assess diabetes distress, especially in patients whose diabetes is poorly controlled. For those who have diabetes-related emotional distress, providing necessary diabetes information, arranging for diabetes self-management improvement program or problem solving therapy, or giving encouragement, are important to alleviate diabetes distress and improving patient outcomes (21, 40).

The strength of our study was that we validated the English version of the DDS-17 among patients with type 2 diabetes of different ethnicity, as compared to previous studies [25, 40, 44, 45]. Although the experience of an illness may be universal, ethnicity and cultural differences may influence how a patient reacts to diabetes and self-treatment; as family dynamics and the responsibilities within a family may differ from one ethnic group to another (6).

A limitation of our study was that participants were recruited via convenience sampling, and only from one center, so they may not be representative of the broader population of patients with Type 2 Diabetes in Malaysia. However, our cohort was representative of the English-speaking patients with diabetes in Malaysia. Another limitation was that we did not include a patient representative in our expert panel when we performed face and content validity. Lastly, if we had increased our sample size from 1:5 to 1:10 for the item to participant ratio, the dimensionality of our instrument would have performed better [46].

## Conclusion

The English version of the DDS-17 was found to be a valid and reliable instrument to assess diabetes distress among type 2 diabetes patients in Malaysia. With the availability of both the English and the Malay versions of the DDS-17, these instruments can now be used in clinical practice among patients who can understand English or Malay, to offer a useful indicator of the effect of diabetes-induced distress during clinic visits, especially for patients with poor glycemic control. This would ensure that these patients are provided the care required.

## Additional file

Additional file 1: The Diabetes Distress Scale (DDS-17). (DOCX 14 kb)

## Abbreviations

DASS-21: Depression Anxiety Stress Scale
DDS-17: Diabetes Distress Scale
DHP-18: Diabetes Health Profile
DSQOLS: Diabetes-specific Quality-of-Life Scale
Hb_A1c_: HaemoglobinA1c
ICC: Intra-class correlation coefficient
PAID: Problem Areas in Diabetes Scale
QSD: Questionnaire on Stress in patients with Diabetes
