# Supplementary material for: The validity and reliability of the English version of the diabetes distress scale for type 2 diabetes patients in Malaysia
Source: BMC Fam Pract. 2017 Feb 20;18:25. doi: 10.1186/s12875-017-0601-9 (PMC5319150; doi:10.1186/s12875-017-0601-9)
Supplement: Additional file 1: — The Diabetes Distress Scale (DDS-17). (DOCX 14 kb) [file 12875_2017_601_MOESM1_ESM.docx]

**Appendix 1: The Diabetes Distress Scale (DDS-17)**

**DIRECTIONS:** Living with diabetes can sometimes be tough. There may be many problems and hassles concerning diabetes and they can vary greatly in severity. Problems may range from minor hassles to major life difficulties. Listed below are 17 potential problem areas that people with diabetes may experience. Consider the degree to which each of the 17 items may have distressed or bothered you DURING THE PAST MONTH and circle the appropriate number.

Please note that we are asking you to indicate the degree to which each item may be bothering you in your life, and NOT whether the item is merely true for you. If you feel that a particular item is not a bother or is not a problem for you, you should circle "1". If it is very bothersome to you, you should circle "6".

|  | **Not a Problem** | **A Slight Problem** | **A Moderate Problem** | **Somewhat Serious Problem** | **A Serious Problem** | **A Very Serious Problem** |
| --- | --- | --- | --- | --- | --- | --- |
| 1. Feeling that my doctor doesn't know enough about diabetes and diabetes care. | 1 | 2 | 3 | 4 | 5 | 6 |
| 2. Feeling that diabetes is taking up too much of my mental and physical energy every day. | 1 | 2 | 3 | 4 | 5 | 6 |
| 3. Not feeling confident in my day-to-day ability to manage diabetes. | 1 | 2 | 3 | 4 | 5 | 6 |
| 4. Feeling angry, scared and/or depressed when I think about living with diabetes. | 1 | 2 | 3 | 4 | 5 | 6 |
| 5. Feeling that my doctor doesn't give me clear enough directions on how to manage my diabetes. | 1 | 2 | 3 | 4 | 5 | 6 |
| 6. Feeling that I am not testing my blood sugars frequently enough. | 1 | 2 | 3 | 4 | 5 | 6 |
| 7. Feeling that I will end up with serious long-term complications, no matter what I do. | 1 | 2 | 3 | 4 | 5 | 6 |

|  | **Not a Problem** | **A Slight Problem** | **A Moderate Problem** | **Somewhat Serious Problem** | **A Serious Problem** | **A Very Serious Problem** |
| --- | --- | --- | --- | --- | --- | --- |
| 8. Feeling that I am often failing with my diabetes routine. | 1 | 2 | 3 | 4 | 5 | 6 |
| 9. Feeling that friends or family  are not supportive enough of  self-care efforts (e.g. planning activities that conflict with my  schedule, encouraging me to  eat the "wrong" foods). | 1 | 2 | 3 | 4 | 5 | 6 |
| 10. Feeling that diabetes controls my life. | 1 | 2 | 3 | 4 | 5 | 6 |
| 11. Feeling that my doctor doesn't take my concerns seriously enough. | 1 | 2 | 3 | 4 | 5 | 6 |
| 12. Feeling that I am not sticking closely enough to a good meal plan. | 1 | 2 | 3 | 4 | 5 | 6 |
| 13. Feeling that friends or family don't appreciate how difficult living with diabetes can be. | 1 | 2 | 3 | 4 | 5 | 6 |
| 14. Feeling overwhelmed by the demands of living with diabetes. | 1 | 2 | 3 | 4 | 5 | 6 |
| 15. Feeling that I don't have a doctor who I can see regularly enough about my diabetes. | 1 | 2 | 3 | 4 | 5 | 6 |
| 16. Not feeling motivated to keep up my diabetes self­ management. | 1 | 2 | 3 | 4 | 5 | 6 |
| 17. Feeling that friends or family don't give me the emotional support that I would like. | 1 | 2 | 3 | 4 | 5 | 6 |
